# High-performing general practice registrars, the gifted and the talented: Helping them to reach their potential

**DOI:** 10.15694/mep.2018.0000093.1

**Published:** 2018-05-07

**Authors:** George Zaharias

**Affiliations:** 1 Royal Australian College of General Practitioners

**Keywords:** High-performing learners, Gifted and talented learners, Theory of learning, Promoting excellence, General practice training

## Abstract

This article was migrated. The article was marked as recommended.

The under-performing medical student or trainee in difficulty, is the subject of extensive discussion in the medical education literature. In contrast, literature on the high-performing medical student or trainee is limited, possibly because high-performers are considered the ‘quiet achievers’ requiring little assistance. High-performers however are diverse and they may also have difficulties with their learning and face particular impediments. Whether quiet achievers or otherwise, high-performers provide challenges to the educator and educational institution alike. This is a discussion paper based largely on the author’s broad experiences in General Practice (GP) training. It references educational theories of giftedness and knowledge management theory in order to provide an understanding of high-performing GP Registrars (GPRs) and the gifted, their attributes, particular challenges and needs. It argues that high-performing GPRs, those having talent or showing potential, should be supported and encouraged to excel. This requires striking the right balance between challenge and support as well as the optimization of: learning, experience, self-reflection and self-actualization. In addition, impediments to learning should be addressed, psychosocial needs met and assistance provided to surmount any disabilities. This is important, not only that high-performing GPRs will aim higher and attain their ultimate potential but also to give impetus to the gifted amongst them, who have special abilities and the potential to make new discoveries, challenge extant ideas and create new paradigms.

## Introduction

The under-performing medical student or the ‘trainee in difficulty’, is the subject of extensive discussion in the literature. ^1, 2, 3, 4^ The high-performing medical learner ^5, 6^ however, receives very little attention in the literature and in practice. This is certainly true of General Practice (GP) training in Australia where processes for managing high-performing GP Registrars (GPRs), or trainees, are not as developed as they might be. To some degree it is because many high-performing GPRs do not stand out. Moreover, they are considered ‘quiet achievers’ who don’t need assistance. Medical educators and supervisors may even feel inadequate because these GPRs are more knowledgeable than they are. High-performing GPRS however do not form a homogeneous group. They have particular learning needs and require support.

This paper has arisen from the author’s experiences in GP training, largely in managing under-performing GPRs but including many encounters with high-performing GPRs, discussions and workshops with supervisors and educators. It presents the author’s perspective on high-performing GPRs and those amongst them who are gifted and talented. As opposed to the high-performing GPR, the gifted and talented can be difficult to identify, nevertheless, they deserve consideration.

As with the literature on high-performers, there is a paucity of literature with respect to gifted adults. However, there is an extensive body of educational theory on giftedness in children and some of this theory is referenced to provide an understanding of gifted and high-performing GPRs. The two share many attributes and this is only natural if we consider that giftedness is at the high end of the spectrum of high-performance.

The author contends that the educational needs of high-performing GPRs should be addressed and they should be encouraged to strive for excellence. Referencing gifted theory once more, along with knowledge management theory, specific recommendations are made for supporting high-performing GPRs. In addressing the needs of the high-performer, those amongst them who are gifted and talented, will be given the impetus to excel and to later go on to progress the profession, assist the community in novel ways and develop new paradigms.

## Educational theories of giftedness

Theory on giftedness is based largely in primary and secondary education. ^7, 8^ There are several definitions and models of giftedness ^7, 8, 9, 10^ and in many respects, the definition depends on the construct. ^11^ Gagné, ^12, 13^ defines it as “the possession of outstanding natural abilities in at least one domain and at a degree that places the individual at the top 10% of age peers”. It is not high intelligence or natural abilities alone. Giftedness arises from the interplay between several factors by which skills are systematically built, namely: outstanding natural abilities; intrapersonal factors; environmental influences; and importantly, chance and maturation. ^12, 13, 14^ While outstanding natural abilities are a prerequisite, it cannot be assumed that a gifted or talented child will invariably become a gifted adult. The positive or negative expression of the various factors, the interplay between them and the ultimate outcome will differ for each individual. ^12, 13, 15^ The role of chance is primary. ^12^ It might be, for example, the difference between missed opportunities and opportunities that come at just the right moment to propel the individual forward.

The gifted are often spoken of as being ‘talented’ but having talent and being gifted are not the same. Talent is the “outstanding mastery of systematically developed abilities” ^12^ and can be acquired, whereas special or natural abilities are essential for giftedness. ^12, 13, 16^ Giftedness also holds the potential for exceptional achievements and it is only from their work that the gifted are identified. ^12, 13^ Support for the gifted is important therefore, because without it, they may not progress as well as they might. ^17^

## Attributes of giftedness

The gifted have attributes which distinguish them from others. They are intrinsically motivated with a pronounced need and drive to excel and be challenged. They have great curiosity and ability to think outside the box, to generate novel solutions and original ideas. They have a high tolerance and preference for ambiguity, the unknown and the contradictory. ^16, 18, 19, 20^

According to Renzulli, ^16, 21^ another principal theorist, giftedness requires intelligence, creativity and motivation. Renzulli prefers the term ‘gifted behaviour’ because giftedness (like talent), might manifest in only one domain rather than across a range of domains. Alternatively, potential for giftedness may be slow to be realized or may not be realized at all. Others may show potential but fail to achieve. ^16, 21^ Conversely, some don’t score highly on IQ tests but nevertheless have natural abilities and eventually are identified as gifted.

Renzulli separates gifted behaviour into schoolhouse giftedness and the creative-productive. ^16^ Schoolhouse giftedness is the ability to score highly in cognitive tests. Medical faculties select from the topmost scorers in entrance exams and most of those selected will be ‘schoolhouse gifted’. Development of schoolhouse giftedness focuses on deductive learning, structured training in the development of thinking processes and the acquisition, storage and retrieval of information. ^16^ This type of learning occurs in medical schools.

Creative-productive learners are more difficult to identify. ^16^ Renzulli refers to Csiszentmihalyi ^22^ who identified three types of creative people: the ‘brilliant’ (having unusual and stimulating thoughts), the ‘personally creative’ (who experience the world in novel ways and make discoveries known only to themselves) and the ‘creative without qualification’. Renzulli believes that identifying and fostering those who are ‘creative without qualification’ is important because they are capable of more than just original ideas. Their work has impact on others and they have the potential to bring about change. ^16, 23^

## The challenges associated with giftedness

Despite the natural abilities, or perhaps because of them, giftedness is associated with psychosocial problems. ^24, 25^ The gifted person may not know, for example, how to function socially. ^19, 26^ Some struggle with perfectionism and pressure to succeed. These pressures may come from themselves, from family members or educators. ^27, 28^ The gifted are often aware that they are different and some develop coping mechanisms to not stand out. Others however cannot manage their behaviour or do not recognize that their behaviour creates conflict. For example, they may be disruptive in learning situations because they don’t receive sufficient stimulation and challenge. ^19, 20, 29^ Some become quickly immersed in a project that is intellectually challenging, only to abandon it once their curiosity has been satisfied. This may be perceived as a failure to follow through. ^27^ The gifted have a unique way of looking at problems and situations and others may have difficulty understanding their perspective and thinking. ^19^ This may be perceived as resistance to teaching or inability to understand, when in actual fact they have understood perfectly. ^27^ Alternatively, their passion for issues that interest them can be so deep that they may be perceived as rigid in their thinking ^20^ or having outlandish ideas. ^19^ They are usually highly sensitive and consequently may not accept negative feedback kindly. The gifted learner’s powerful drive for self actualization may also result in conflict when requirements and regulations run counter to what they consider important. ^20, 27^

## The “twice-exceptional” learner

The “twice-exceptional” learner is a term applied to the gifted learner who also has a disability. Giftedness can co-exist with any disability ^25^ although the term is often applied to those having a learning disability (ADHD being the prime example). ^25, 30^ Twice-exceptional learners are identified from the unusual discrepancies between their strengths and weaknesses ^27^ and this has been the author’s experience in managing underperforming GPRs. The strengths of the twice-exceptional learner include: a superior vocabulary; creativity; resourcefulness; curiosity; imagination; and questioning. They have problem-solving abilities, advanced ideas and opinions, a wide range of interests and/or a special talent or consuming interest. Their behaviour can be challenging because they are easily frustrated, stubborn, manipulative, opinionated, argumentative, highly sensitive to criticism, inconsistent and disorganized. ^31^ The challenges presented by a twice-exceptional learner can be complex because they arise from their giftedness and their disability. For some, their disability provides the impetus to strive and succeed while for others, their disability is an impediment. Others will be considered average and their high-performing abilities unrecognized because they compensate for their weaknesses. ^32^

## High-performing and gifted GPRs

There is no accepted definition for high-performing GPRs. From observations, from discussion with supervisors and medical educators, high performing GPRs are considered to be: above average in intelligence, their knowledge base and clinical capability. Often, they are performing beyond the level expected for their stage of training. They are generally considered as having:

• Excellent clinical skills generally, but notably, excellent communication skills; good bedside manner; empathy; good interpersonal and team skills; good organizational and time management skills.
• Motivation to learn, achieve and be challenged; self-direction; ability to identify their learning needs readily, to translate theory into practice and learn readily from experience.
• Well developed cognitive abilities and clinical reasoning skills; ability to take a broad perspective; fresh ideas and a sixth sense; ability to be reflective and insightful, recognising and acknowledging their limitations.
• High emotional intelligence and a mature approach to work and life; tendency to be perfectionists with high standards; confident, often quietly so.

As already noted, these are also attributes of the gifted. ^6, 18, 19, 20^ Educational theory highlights the complexity of giftedness ^6, 19^ and high-performing GPRs are similarly complex in their attributes as well as their psychosocial needs. Furthermore, their attributes can be both positive and negative. ^20^ For example, their knowledge base may be exceptional but narrowly focused and they may have difficulty imparting that knowledge. This would be problematic when trying to present information clearly and concisely to patients. Knowledge may be strong in some areas and weak in others because they focus on what interests them or what they consider relevant. Consequently, if they are not interested in something, they may not be motivated to act. This can manifest as poor work ethic. For example, they might be happy to see many patients at the clinic but have no interest in doing home visits.

Also, not all high-performers are humble or modest. On the contrary, they can be arrogant and condescending towards others because they know that they are intelligent and capable. This can also manifest as overconfidence and attempting of tasks outside their capability. They may be unwilling to admit mistakes or accept criticism. They may even seek to hide their weaknesses and mistakes by not asking questions or engaging in case discussion.

Furthermore, their belief in something can be so strong and even though their belief might be correct, they are perceived as rigid and lacking insight. Their perfectionism and obsessive tendencies can work against them, making them highly anxious. They may set unreasonable goals for themselves and may be very self-critical, almost to the point of being self-destructive because they don’t want to fall short of expectations, their own or others’. They may try to do too much and then appear to have difficulty with prioritizing and time management. They may worry excessively about missing something or about medicolegal ramifications that they write copious notes, staying back late to complete them, or calling the patient after hours to check whether anything has gone awry. They can become very distressed, especially when things don’t go as expected and they may therefore be perceived as emotionally needy or petulant. Arrogance and petulance do not make for good interpersonal relationships and teamwork.

At one extreme then are the highly intelligent, motivated and capable GPRs who are left to their own devices because of the belief that “there is nothing to teach them” ^5^ or because the supervisor is daunted by their abilites. ^5, 6^ At the other extreme are the GPRs, equally capable, who present challenges because of their attitude and behaviour. Amongst these are the twice-exceptional. In between the extremes is a diverse spectrum of strengths, weaknesses behaviours and needs. Knowing how to address the individual needs of all these highly capable GPRs is indeed a challenge.

## Recommendations for managing high-performing and gifted GPRs

Before looking at how the needs of high-performing and gifted GPRs can be addressed, it is worth looking at knowledge management theory. Snowden has described a model for how knowledge is used and decisions made. ^33, 34^ It is a model that is applicable to medical education. ^35^

Four domains are described: known, knowable, complex and chaos. The *known* ^33, 34^ is the domain of the medical student, ^35^ of well-defined processes, where decision-making is relatively simple. While a certain level of knowledge is requisite, there is not much processing and very little challenge.

In the *knowable*, ^33, 34^ the problem or the solution is not clearly defined and information requires more processing. Basic problem solving skills are utilised and assistance obtained from someone more experienced. This is the domain of the postgraduate trainee under supervision. ^35^

In the *complex*, ^33, 34^ many variables with differing relationships have to be considered. This is the domain of the experienced GP, of narratives, multiple morbidities, chronic complex disease, unusual presentations and undifferentiated illness. ^35^ Decision-making is context dependent and intuitive, ^36^ requiring practice-specific skills. ^37, 38^

GPRs eventually have to be able to manage chronic complex problems, to think intuitively and work independently. In other words, be able to function in the complex domain. High-performing GPRs generally, are already functioning well in the knowable domain. They rise to the challenges of the complex domain and some may already be functioning well in it. They seem to do everything more easily. ^6^ They have a broader knowledge base and learn new skills very quickly. They may lack in clinical experience but they certainly learn quickly from it. They are able to think more widely and intuitively and are willing to be challenged and to tackle complex problems. Some may argue that they don’t need to be challenged further but should be allowed to broaden their skills and gain more experience. High-performing GPRs however want more than this. It is only logical therefore to challenge them and to support them to aim higher.

High-performing GPRs therefore can be introduced to the domain of *chaos,* ^33, 34^ the domain of unchartered waters, research and new discoveries. This domain requires people with special abilities, abilities which they themselves may not be able to understand or explain. This domain is for GPRs who are gifted, having special talents or who are creative.

As has been noted earlier, the gifted may not be truly identified until much later in their lives. ^11, 12^ Also, because of the spectrum of presentations, it is easier to identify someone as being high-performing or having talent and potential, rather than gifted per se. Assisting the high-performer to further develop their strengths and areas of special interest is one starting point. (Cases 1, 2) But even high-performing GPRs will have weaknesses as regards knowledge and clinical skills. Sometimes they are identified as under-performing because of the nature of their weaknesses or because of a disability. Whatever the nature of the issues, they have to be addressed. (Cases 3, 4)

Challenge is essential for learning and for competence to develop. Knowing how much challenge and support is required is not always easy. ^39^ Providing the right balance in a group setting is difficult. ^39^ For GPRs under supervision it is much easier. Under-performing GPRs require more support and a lesser degree of challenge whereas high-performers require a greater degree of challenge and a lower level of support. ^22, 39, 40^

What high-performers need by way of support is: a challenging education, high level opportunities and someone who believes in them. ^26^ Instruction should be inquiry oriented using strategies such as problem based learning and Socratic questioning so that learners can create their own understanding of the subject in such a way that it encourages the application of appropriate processes to new and complex situations. ^9, 15^ The educator may need to motivate the learner because some learners aren’t necessarily self-motivating or may get bored easily. They should be encouraged to examine material from multiple perspectives. All assessments and feedback should be formative, ^14^ capitalizing on strengths and minimizing weaknesses. ^9^

The learning environment should be supportive and excellence encouraged. High-performers require opportunities to be creative and to develop their interests. ^41, 42^ (Cases 1, 2) They should also be allowed to present and defend their work. The development of creative-productive learning focuses on thinking processes and the application of knowledge in an integrated, inductive and problem oriented manner which allows the learner to be inquiring and self-determining. ^16, 21^ This type of learning is more readily facilitated in the GP training context.

The psycho-social needs of high-performers are just as important as their intellectual needs. ^19, 24^ Assisting them to overcome difficulties created by a disability is vital, for their personal well being as well as their personal growth. ^28^ (Cases 4, 5)

The principles for managing high-performers are: to optimize learning, experience, self-reflection and self-actualization. ^28^ [ Appendix I] This entails:

• Challenge: to manage more difficult and more complex problems (to operate effectively in the *complex* domain); to strive higher (to consider the domain of *chaos*); to realise their aspirations and potential.
• Support: direction and guidance rather than instruction; development of strengths; addressing areas of weakness; addressing psychosocial needs; overcoming impediments due to disability; promoting self-reflection and insight.

## Concluding remarks

This paper does not purport to provide all the answers on the subject of high-performing GPRs, the gifted and the talented. It has presented thoughts on a topic that requires much more discussion and certainly much more research.

All GPRs are highly intelligent because of the selection processes that they have been through. The challenge for GP training is to provide them with the requisite skills so that they will be prepared to meet the increasing challenges and complexities of General Practice. Most GPRs will attain the requisite level. Many GPRs, who are high-performing and talented, will seek out challenges for themselves and will surpass that level without much assistance. However, leaving them to their own devices is not appropriate because they can falter. Supporting high-performers, addressing their needs, assisting them to overcome any obstacles and disabilities, increases their chances of reaching their ultimate potential and becoming fulfilled in their professional lives.

High-performers, those having talent and potential should be identified. They should be challenged to strive for excellence, not only for their personal benefit but also the benefit of the community and the profession. The gifted among them, having been given the impetus, will achieve in exceptional ways. Their drive and creativity will lead them to make new discoveries, to challenge extant ideas and create new paradigms.

## Take Home Messages

High-performers:

• can and should be identified
• can sometimes struggle with their learning
• should be challenged to strive for excellence

The gifted and talented:

• aren’t always easily identified
• are generally found amongst the high-performers
• when given the impetus, will achieve in exceptional ways

## Notes On Contributors

Dr George Zaharias is a General Practitioner and Medical Educator, in Melbourne, Australia. His field of expertise is remediation, having managed the remediation of Registrars in a General Practice training program for over 10 years. In his current role with the Royal Australian College of General Practitioners, his responsibilities relate to the remediation of General Practice Registrars and General Practitioners as well as assisting General Practitioners with returning to work.

## Appendices

### Appendix I. Recommendations for the enhancement of high-performing GPRs (adapted from Morgan, ^5^ Seehusen & Miser ^6^, Ramani & Leinster ^39^)

**Table:**

*Methods for enhancing clinical skills:* • Provide more challenge: consultations with complex and challenging presentations, home visits and nursing home patients • Teach: complex care coordination; the broader impact of illness on family and others; a narrative approach • Role-play challenging consultations • Discuss: diagnostic dilemmas; alternative clinical scenarios beyond the immediate patient (“hypotheticals”) • Direct observation of consultations with feedback • Debrief of video-recorded consultations *Guiding principles for the educator:* • Provide a stimulating learning environment and intellectual challenges • Take a collaborative approach to teaching and learning; consult together • Contextualise teaching with the wisdom of clinical experience • Blend the art with the science • Role modeling: recognize limitations; admit mistakes; leadership skills; life-long learning; self-care • Promote excellence *For the GPR as an individual:* • Explicitly acknowledge them as highly performing • Provide opportunities for them to engage with other high-performers • Develop their strengths • Encourage exploration of specific interests • Encourage creativity • Encourage development of self-reflective powers (awareness of strengths, limitations and behaviour) • Promote self-care • Develop their powers of resilience

### Appendix II. Case studies

**Table:**

**Case 1: “Manuel”** Manuel is a high performing GP registrar. His supervisor enjoys teaching him because of the intellectual challenges that Manuel provides even though they have widely differing viewpoints. Manuel believes strongly in evidence-based medicine while his supervisor brings the wisdom of his many years of clinical experience. Manuel values the relationship with his supervisor because he is treated as an equal and their exchanges are always two-way learning opportunities. Manuel has a creative side. He produces and posts on-line technically masterful and engaging educational videos. His supervisor is very supportive of this. Manuel fails his qualifying exam and later admits that he did not study because he was too distracted by his creative pursuits. **Case 2: “Sandra”** Sandra is in the first year of her GP training. Her supervisor identifies her to be highly functioning. She already has very good clinical skills, is strongly self confident and does not shy away from heartsink patients. Her supervisor, unsure of what more she can do, asks Sandra how she can assist her with her learning. Sandra says that she is very keen to develop her mental health skills, particularly with adolescents, but doesn’t know how to go about it. Mental health is not her supervisor’s strength, however, together, they look at ways that will enable Sandra to develop her skills (other doctors in the clinic who manage such problems; engaging with a mental health service, psychiatrist or psychologist; courses for advanced mental health skills; ways in which she can receive supervision when managing adolescent mental health problems). **Case 3: “Belinda”** Belinda has just commenced GP training. Her supervisor is both concerned and perplexed. In tutorials, Belinda appears to be very knowledgeable and capable. In practice however, Belinda is slow with her consultations and asks questions about every patient that she sees. The supervisor doesn’t understand why Belinda does this when invariably she finds that Belinda has acted appropriately. Direct observation of Belinda’s consultations is conducted where it is noted that she is very meticulous in her history taking and physical examination and is able to arrive at an appropriate diagnosis. However, Belinda worries excessively about missing something potentially serious and transfers her anxiety onto the patient by focusing on quite unlikely diagnoses and possibilities as well as providing lengthy explanations and more information than necessary. Discussion with Belinda encourages her to consider the consultation from the patient’s perspective and how the patient might feel with respect to the information that is provided. Belinda readily identifies how she is causing unnecessary confusion and anxiety for the patient. Discussion then focuses on how to entertain a reasonable differential, exclude possible serious diagnoses and provide the patient with the necessary, concise information, without causing unnecessary anxiety for herself and at the same time, not being alarmist. Belinda is able to put these strategies in place very easily and progresses quite well. Later, the supervisor identifies Belinda as high-performing. **Case 4: “William”** William’s supervisor has noted that William’s consultations are unnecessarily lengthy for his stage of training and believes that he has poor consulting skills. He has also noted that William is socially awkward. Direct observation of William’s consultations is conducted. It is observed that he creates good rapport with patients. History taking and physical examination are satisfactorily done and he arrives at an appropriate diagnosis. However, he is excessively anxious and this impedes the delivery of the management because he hesitates a lot, takes extraordinarily long to provide instructions and information and then to bring the consultation to a close. In giving feedback to William, when mention is made of his manifest anxiety, he divulges that he has Asperger’s. A learning plan is drawn up to assist William. He is provided with regular extra teaching time focusing formulating and effectively and efficiently delivering a tailored management plan. The supervisor makes arrangements for William to see a broader range of patient presentations, including chronic disease. William is also advised to seek professional assistance to manage his anxiety and any other personal issues. William is a willing learner and applies himself to improving his skills. William also consults his psychiatrist. William demonstrates satisfactory improvement and completes his training. He continues working in general practice for a short while and even though he is well supported by his colleagues, he finds work very stressful and eventually decides to pursue a different career. **Case 5: “Damian”** Damian has almost completed his GP training and has been progressing well and without incident. His current supervisor notices that Damian tends to stay in his room when not seeing patients and does not engage with colleagues and staff. Without explanation, Damian is suddenly frequently absent from work (days at a time). The supervisor confronts Damian about this. Damian says that he has been experiencing some personal stresses lately but there is no cause for concern. From the tone of the conversation, the supervisor surmises that Damian is depressed. Damian concedes that this is true. The supervisor urges Damian to take time off and consult his treating doctor. Damian complies. He returns, having recovered from his depression, to complete his training. Damian joins a group general practice in a lower socio-economic area. He proves to be a very capable GP. He takes on a special project, to develop and co-ordinate a program for addressing the mental health concerns of adolescents in the community.
