# Supplementary material for: High-performing general practice registrars, the gifted and the talented: Helping them to reach their potential
Source: MedEdPublish (2016). 2018 May 7;7:93. [Version 1] doi: 10.15694/mep.2018.0000093.1 (PMC10711976; doi:10.15694/mep.2018.0000093.1)
Supplement: Peer review history

# Reviewer report: Reviewer response for version 1

**DOI:** 10.21956/mep.19569.r29032

**Published:** 2018-05-11

**Authors:** Johnny Lyon Maris

**Affiliations:** 1 Southampton GP Education Unit

This review has been migrated. The reviewer awarded 5 stars out of 5 This is a well written, researched and evidenced paper. It is an important and frequently neglected area, where the majority of energy is put into poor performance rather than giftedness. The phrase; They are usually highly sensitive and consequently may not accept negative feedback kindly, I find especially important for educational supervisors to realise. The Snowden model I find entirely appropriate for this group and like this framework to help guide the development of these learners from complex to chaos. Thankyou for this paper it has helped me develop my thoughts in this area.

Reviewer Expertise:

NA

No decision status is available

# Reviewer report: Reviewer response for version 1

**DOI:** 10.21956/mep.19569.r29030

**Published:** 2018-05-09

**Authors:** Julie Hunt

**Affiliations:** 1 Lincoln Memorial University

This review has been migrated. The reviewer awarded 4 stars out of 5 The author's exploration of high-performing GPRs is helpful in understanding the range of learners that come into clinical placements. As a clinical supervisor, I'm glad for the reminder that high performers still require mentorship. The appendices were helpful in making the article's content actionable. I also appreciate the author's description of the paradox of high performance and giftedness actually impairing some skills and traits required for success in clinical practice.

Reviewer Expertise:

NA

No decision status is available

# Reviewer report: Reviewer response for version 1

**DOI:** 10.21956/mep.19569.r29031

**Published:** 2018-05-08

**Authors:** Varsha Patel

**Affiliations:** 1 NHL Municipal Medical College

This review has been migrated. The reviewer awarded 4 stars out of 5 Well written. I appreciate the focus on unusual subject. Often the gifted students can't perform well due to lack of identification and support by mentor / teacher.

Reviewer Expertise:

NA

No decision status is available
